# Acceleration of wound healing in acute full-thickness skin wounds using a collagen-binding peptide with an affinity for MSCs

**DOI:** 10.4103/2321-3868.143623

**Published:** 2014-10-25

**Authors:** Huili Wang, Xin Yan, Liangyun Shen, Shiyan Li, Yue Lin, Shuqin Wang, Xiang Lin Hou, Chunying Shi, Yun Yang, Jianwu Dai, Qian Tan

**Affiliations:** 1 Department of Burns and Plastic Surgery, The Affiliated Drum Tower Hospital of Nanjing University Medical School, Nanjing, 210008 China; 2 Institute of Genetics and Developmental Biology, Chinese Academy of Sciences, Zhongguancun, Beijing, China

**Keywords:** Mesenchymal stem cells, skin wound healing, Collagen-binding domain, mesenchymal stem cells affinity peptide

## Abstract

Mesenchymal stem cells (MSCs) have been accepted as a promising cell source in tissue repair and regeneration. However, the inability to enrich MSCs in target areas limits their wide application. As a result, it has been a major goal to induce MSCs to be abundantly and specifically recruited to the injury site. In this study, a peptide with a specific affinity for MSCs (E7 peptide) was immobilized to a collagen scaffold via a collagen-binding domain (CBD) to construct a functional collagen scaffold. In addition, the hypothesis that this method could recruit MSCs specifically was evaluated in a porcine model. *In vivo* investigations indicated that due to the immunore-action, the CBD-MSC-peptide collagen scaffold enhanced MSC adhesion and infiltration and promoted wound healing. At day 7 after surgery, we found more infiltrating cells and capillaries in the Collagen/CBD-E7 peptide group compared to the Scaffold group. At day 14, 21 and 28, a faster healing process was observed in the Collagen/CBD-E7 peptide group, with significant differences compared with the other groups (*P* < 0.05, *P* < 0.01). The results demonstrate the potential use of targeted therapy to rapidly heal skin wounds.

## Introduction

Massive full-thickness wounds caused by extensive burns or high-energy trauma are common in the clinical, but treatment for these emergencies is limited and largely ineffective.[1] In recent years, stem cell therapy has been considered as an effective method of tissue regeneration.[2] Mesenchymal stem cells (MSCs) are stromal cells that exhibit the characteristics of self-renewal and multi-lineage differentiation[3] and which have been accepted as a promising cell source in tissue repair and regeneration.[4] Studies have shown that during wound repair, ischemia or cancer invasion, MSCs mobilize to the circulation, recruit to the injury site and promote tissue repair.[5,6] However, the stem cells that are recruited to the wound site for tissue regeneration are insufficient.[7] As a result, broader mobilization and recruitment of MSCs specifically to the injury site has been a major area of study.

**Table 1:** 

Access this article online	
**Quick Response Code**:	**Website**: www.burnstrauma.com
	**DOI**: 10.4103/2321-3868.143623

Recently, a novel peptide (E7 peptide, “EPLQLKM”) was reported to have high, specific affinity to MSCs. When covalently conjugating to a synthetic polycaprolactone (PCL) mesh, this peptide significantly mobilized and recruited autologous MSC to PCL *in vivo*.[4] Compared to synthetic materials, collagen has commonly been used in wound repair and skin regeneration due to its weak antigenicity and excellent biocompatibility.[8] Binding of a specific stem cell affinity protein to a biomaterial has also successfully been applied to the regeneration of damaged myocardium.[6] However, few studies have put such a method to use in the treatment of massive full-thickness wounds.[9]

As described previously, the collagen-binding domain (CBD) shows great affinity to collagen I and can maintain growth factor activity both *in vitro* and *in vivo*.[10,11] To this end, we produced a fusion protein (CBD-MSC-peptide) consisting of the MSC affinity peptide (MSC-peptide) and a CBD. This protein was able to specifically bind to collagen I and allowed the MSC-peptide to bind to collagen. We hypothesized that due to the immune reaction induced by the scaffold, MSC cells would migrate to the target region and promote both wound healing and skin regeneration. The hypothesis was evaluated in a porcine full-thickness defect model.

## Materials and methods

### Preparation of collagen/CBD-E7 peptide functional scaffolds

**Figure 1:**
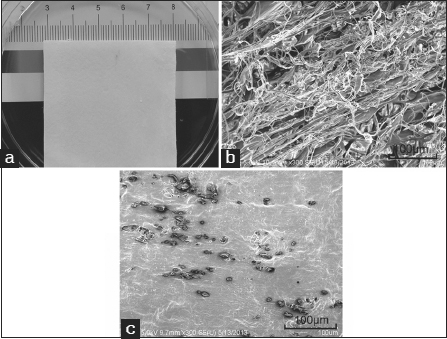
Collagen scaffold morphology. (a) Macroscopic view of the collagen scaffold; (b) Scanning electron microscopy (SEM) image of the collagen scaffold. Scale bar =100 µm; (c) SEM image of the collagen scaffold conjugated with E7 peptide. Scale bar =100 µm.

The collagen membrane was prepared as described previously.[6] It was cut into 5.0 × 5.0 cm squares with a thickness of 1.0 mm [Figure 1a]. CBD-E7 peptides were synthesized through solid-phase peptide synthesis using Fmoc Chemistry (Scilight-Peptide Inc., Beijing, China). Next, 250 µg of CBD-E7 peptides were dissolved in 100 µl phosphate-buffered saline (PBS) and added to the collagen membrane. The surface morphology of the collagen scaffold before and after treatment was observed by scanning electron microscopy (SEM) (model S-2500, Hitachi, Japan) [Figure 1b and c].

### Animal models

Six domestic pigs weighing between 20 and 25 kg were used for implantation. There was no injection of granulocyteolony stimulating factor (G-CSF) before the skin defect model was induced. After the administration of anesthesia by propofol, the dorsal hair was shaved. Six full-thickness wounds (3 incisions on each side, 5.0 × 5.0 cm) were made on the dorsum down to the level of subcutaneous deep fascia, and different collagen membranes were affixed to the defects with 4−0 silk suture.

Total 36 wounds were randomly divided into 3 groups: A control group (Control, *n* = 2 × 6), a collagen group (Collagen, *n* = 2 × 6), and a Collagen/CBD-E7 peptide group (Collagen/CBD-E7 peptide, *n* = 2 × 6).

After the operation, the pigs were housed in separate cages. All animals received intramuscular penicillin for 3 days after surgery. All of the pigs survived during the experiments. All of the animals and experimental procedures were approved by the local Institutional Animal Care committee. Chinese Ministry of Public Health (CMPH) guidelines for the care and use of laboratory animals were abided by during all experiments.

### Analysis of cells on the material

Three days after the surgery, 3 animals were sacrificed. Samples of the material were removed, and a portion of the membranes (1.0 × 1.0 cm) were excised for cell infiltration analysis. The cells retained on the collagen were stained with Hoechst 33342 (2 mg/ml, Sigma, USA) for 15 min. After washing them three times, photos were taken.

Part of the deep fascia (1.0 × 1.0 cm) that included the collagen membranes was excised. After fixation in 10% formalin, 5 µm paraffin sections were stained by hematoxylin and eosin (HE) for analysis of the inflammatory reaction and cell infiltration.

In a separate experiment, the remaining collagen was digested using collagenase type I, and the cells that were retained on the materials were collected. The cells were cultured in standard liquid culture medium containing DMEM-F12, 10% FBS, 100 U/ml penicillin, 100 mg/ml streptomycin at 37° C in a 5% CO_2_ atmosphere to the second passage. Then, the cells were prepared for fluorescence activated cell sorting (FACS) analysis to determine whether the materials were enriched in cells that with stem cell characteristics.

### Measurement of healing rate in a porcine model

The wound area was examined and photographed by digital camera (Canon EOS 550D) on day 7, 14, 21 and 28 post-surgery. The area of the unhealed wound was measured using Photoshop software in order to calculate the healing rates (Adobe Photoshop 7.0). The following equation was used: Healing rate = [(original size — non-healing area)/original area] × 100%.[11]

### Vascularization evaluation

At day 7 after operation, a portion of the wound samples (1.0 × 1.0 cm) that included the materials was excised. After fixation in 10% formalin, 5 µm paraffin sections were stained with HE, and immunohistochemical (IHC) analysis was performed for analysis of cell proliferation and blood vessel formation. An anti-von Willebrand factor antibody (vWF, 1:800, Abcam, USA) was used to evaluate the degree of vascularization in the wound granulation tissues. Two independent observers quantified the number of blood vessels for each section, and counts were made for six random selected areas at 200× magnification level in a blinded fashion.

### Statistical analysis

Statistics were calculated with SPSS computer software for Windows (version 13.0, SPSS Inc., Chicago, IL). The values were expressed as means ± standard deviation (SD). Significant differences between the groups were determined by one-way analysis of variance (ANOVA). The least-significance-difference method (LSD) or Tamhane’s T2 test was used for post-hoc multiple comparisons for equal or unequal variances. A probability value (*P*) of less than 0.05 was considered to be statistically significant. Statistically significant values were defined as *P < 0.05 and **P < 0.01.

## Results

### The collagen scaffold

The structure of the collagen scaffold was observed by SEM. Figure 1b and c show the appearance of collagen scaffold before and after treatment with E7 peptide. When conjugated with the E7 peptide, the interconnected pores of collagen scaffold were mostly filled.

### Cell analysis of collagen scaffolds

**Figure 2:**
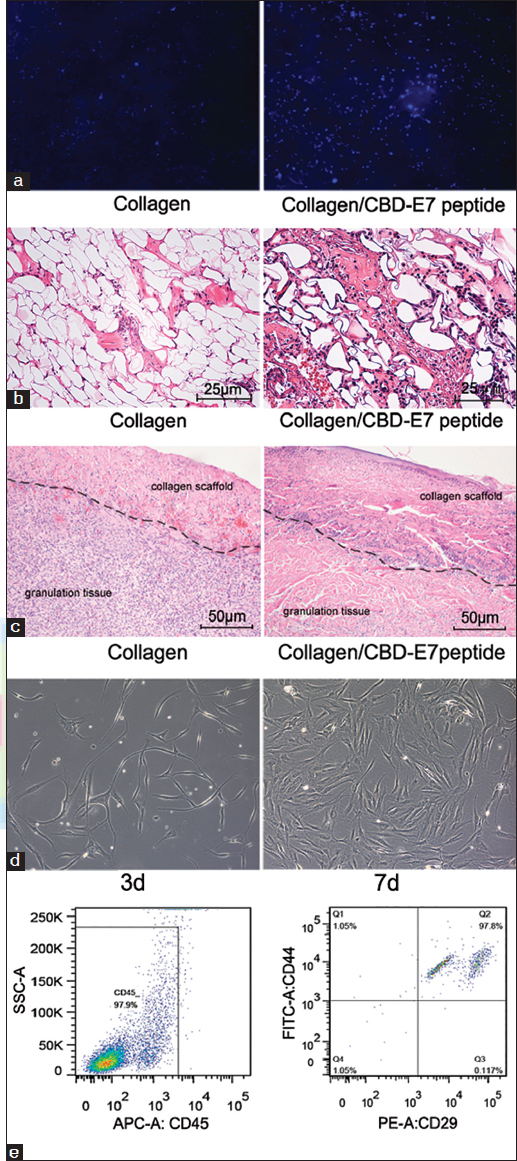
Cellularization of collagen scaffolds. (a) Hoechst 33342 staining of the cells retained on the collagen with different treatments; (b) Cellular distribution in the deep fascia. The number of the cells infiltrated in the deep fascia in the collagen-binding domain (CBD)/peptide group was significantly higher than that in the Scaffold group (per field); (c) Histological evaluations of specimen tissues implanted with collagen scaffolds (left) and collagen/CBD-E7 peptide at day 7 post-surgery. The black curve shows the border between the material and the granulation tissue. (hematoxylin and eosin stain, ×200). (d) Cells derived from CBD-E7 Collagen scaffold at day 3 exhibit characteristic phenotypes of Mesenchymal stem cells (MSCs) (fibroblast-like growth and clumped together in a “swirl” pattern). (e) Fluorescence activated cell sorting (FACS) analysis of the cells remaining on the collagen scaffold.

At day 3 post-surgery, a portion of the materials was removed, and there were significantly more cells retained on the CBD-E7 collagen scaffold than on collagen scaffold [Figure 2a]. A greater number of cells were recruited to the deep fascia in the Collagen/CBD-E7 peptide group, whereas in the Collagen group, the distribution of the cells was sporadic [Figure 2b].

At 7 day after surgery, a sample of granulation tissue with the tested materials was removed and evaluated by HE staining. The scaffold appeared similar to collagen fiber, and more cells had infiltrated in the Collagen/CBD-E7 peptide group [Figure 2c].

The cells enriched on the collagen that were to be used for culture analysis were obtained by digesting the remaining collagen. Three days later, these cells exhibited fibroblast-like growth and clumped together in a “swirl” pattern at day 7 [Figure 2d].

The cultured cells were obtained for molecular phenotype analysis. As demonstrated in Figure 2e, the cells were identified as CD45 negative, CD29 positive and CD44 positive, and thus were characterized as MSCs.

### Wound healing rate

**Figure 3:**
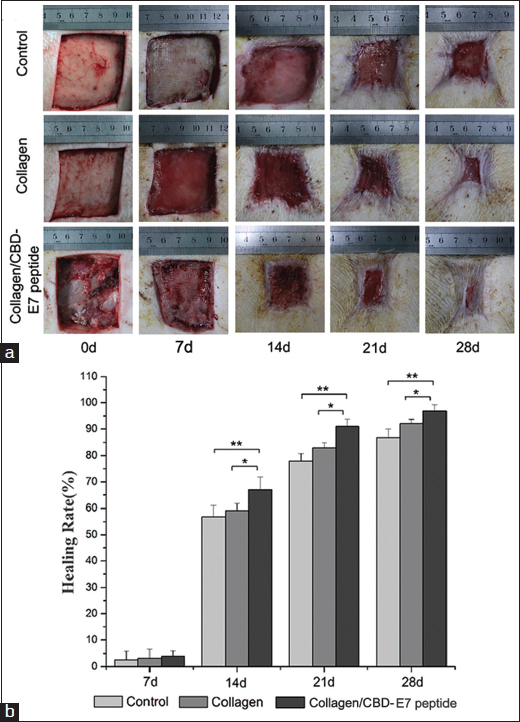
Wound size measurements at different time points. (a) The appearances of the wounds in the different groups at day 0, 7, 14, 21 and 28; (b) The statistical analysis of the healing rate in the different groups. 7 d: Collagen/CBD-E7 peptide: 3.96 ± 1.9%, Collagen: 3.15 ± 3.3%, Control: 2.52 ± 3.1%; 14 d: Collagen/CBD-E7 peptide: 67.04 ± 4.8%, Collagen: 59.11 ± 2.7%, Control: 56.76 ± 4.3%; 21 d: Collagen/CBD-E7 peptide: 90.96 ± 2.7%, Collagen: 82.96 ± 1.92%, Control: 77.98 ± 2.8%; 28 d: Collagen/CBD-E7 peptide: 96.90 ± 2.3%, Collagen: 92.05 ± 1.6%, Control: 86.93 ± 3.1%. The data are presented as the mean ± SD. **P < 0.01, *P < 0.05. CBD = collagen-binding domain. SD = standard deviation.

Seven days after surgery, the healing rates of the 3 groups were not significantly different (*P* > 0.05). However, after 14 days of treatment, the Collagen/CBD-E7 peptide group showed the highest rate of healing among the 3 groups (*P* < 0.05, *P* < 0.01). Moreover, a significantly more rapid wound healing process was observed in Collagen/CBD-E7 peptide group at day 21 and 28 than was found for the other groups (*P* < 0.05, *P* < 0.01) [Figure 3]. In addition, the wound-healing rate was significantly higher in the Collagen group compared to the control group after 14 days (*P* < 0.05).

### Angiogenesis induction by the collagen/CBD-E7 peptide scaffold

**Figure 4:**
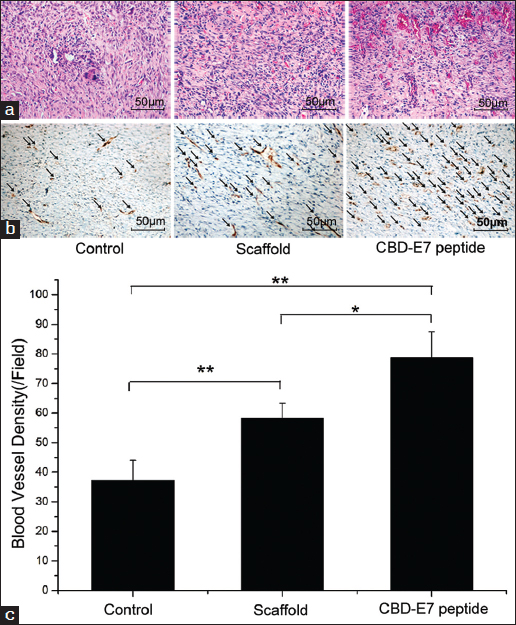
Hematoxylin and eosin (HE) and immunohistochemical (IHC) staining for capillary vessel formation in skin wounds 7 days after surgery (*n* = 6). (a) HE staining. (b) The degree of vascularization in the wound granulation tissues. The arrows indicate blood vessels. (c) The statistical analysis of the blood vessel density. The data are presented as the mean ± SD**P < 0.01. SD = standard deviation.

Capillaries were detected by immunostaining using an anti-vWF antibody. In wound granulation tissue, the capillary density in the Collagen/CBD-E7 peptide group (78.70 ± 8.77) was significantly higher than that of Collagen (58.35 ± 5.03) and control groups (37.30 ± 6.73) [Figure 4b and c], which was consistent with the evidence provided by HE staining [Figure 4a]. It has been shown that the infiltration and migration of cells, especially endothelial cells, are important for angiogenesis.[12] It is possible that a subset of the endothelial cells or hematopoietic stem cells could also be enriched at target site, increasing blood vessel formation around the wound, improving blood perfusion, and thereby accelerating the wound healing process.

## Discussion

Wound healing is one of the basic problems in the surgical field, lacking effective clinical treatment. In most patients, partial skin loss can be managed by closure with remaining local skin[13]; however, for patients with extensive full-thickness injury more than 4 cm in diameter, the incomplete skin cannot regenerate itself spontaneously without a graft.[14]

Stem cell therapy has emerged as a potential therapeutic strategy for full-thickness wound repair.[2] Autologous MSCs are among the most promising cell types, exhibiting the characteristics of self-renewal, multi-lineage differentiation and low immunogenicity.[3] Long-term studies have shown that the perivascular niche could be a location in which MSC-like cells reside.[15,16] Although MSCs can migrate to the injury sites during wound repair, ischemia or cancer invasion, the cells that are recruited for tissue regeneration are insufficient.[17]

In this study, we hypothesized that the Collagen/CBD-E7 peptide group could recruit MSCs *in vivo*. Furthermore, it was proposed that the functional scaffold could promote overall wound repair when used to treat of skin injury. At day 3 *in vivo*, the cell numbers in the designed material and deep fascia reflected the early tendency of cell migration and the biocompatibility of the scaffold [Figure 2a and b]. We deduced that Collagen/CBD-E7 peptide could enhance the recruitment of stem cells to the scaffold and that the scaffold provided a proper environment for cell survival and the subsequent cascading injury response. As is well known, neovascularization is an essential step in the complicated wound healing process.[2] At day 7 after surgery, we found more cells and capillaries infiltrated in the Collagen/CBD-E7 peptide group compared to the Collagen group.

At day 7, 14, 21 and 28 post-surgery, the wound area was examined and photographed for wound healing analysis. Seven days after surgery, the healing rate between the three groups showed no statistically significant difference (*P* > 0.05) [Figure 3]. We deduced that the stem cells did not exhibit their pluripotent potential in this early part of the process. Subsequently, more rapid healing of wounds was observed in the Collagen/CBD-E7 peptide group at day 14, 21 and 28 post-surgery, with significant difference with the other groups (*P* < 0.05, *P* < 0.01) [Figure 3]. This result indicated that the mobilized MSCs could migrate to the scaffold, shorten the healing time and improve wound repair via their multi-lineage differentiation capabilities. Moreover, the 3D collagen scaffold, which was also important for the survival and function of the stem cells in the injury sites, might facilitate cell-scaffold interactions, including migration, proliferation and differentiation.

The MSCs’ specific affinity for the E7 peptide has been successfully used in MSC-homing research.[4] These studies have revealed that the specific binding of E7 to MSCs could induce autologous MSC recruitment in response to trauma or ischemia. A suitable microenvironment is also important for cell migration and tissue regeneration.[2] Collagen is regarded as one of the most useful biomaterials for its excellent biocompatibility and biodegradability, and it is widely used in tissue engineering.[11] As is shown in Figure 1b and c by SEM, the collagen scaffold was porous before treatment. When conjugated with the MSC-binding E7 peptide, the interconnected pores were generally filled, which was important for cell immigration.

It has been considered that CBD technology has the advantage of both durability and specificity. In this study, using the collagen scaffold to bind E7 peptide, which has a specific affinity for MSC, resulted in the capture of autologous MSCs and the acceleration of injured tissue vascularization. As is shown in Figure 4, the blood vessel density in the Collagen/CBD-E7 peptide group was much higher than in the other groups. We concluded that the bio-composite could promote vascularization and, thereby, the subsequent rapid tissue regeneration. As is well known, MSCs are positive for CD29, CD90, CD44, and CD105 but negative for CD34 and CD45.[3,7] In this study, adhered cells on the scaffolds were characterized as MSCs (CD45^−^, CD29^+^, CD44^+^), as were verified by FACS. The results indicated that MSCs played a significant role in promoting wound healing and skin regeneration.

Although the Collagen/CBD-E7 peptide scaffold was effective in MSC recruitment and wound healing, certain limitations remained. First, more samples should be investigated. Second, the E7 peptide (“although the Collagen/CBD-E7 peptide scaffold was effective in MSC rein rabbit models, in which it significantly enhanced autologous MSC recruitment.[4] In this study, we constructed an E7 peptide-based functional collagen scaffold to recruit autologous MSCs in a porcine skin model for wound healing at porcine models. No immune rejection and risk of tumor was observed; however, further studies are required to confirm the clinical efficiency and safety profile of this capture system. Lastly, further research is needed on skin and cutaneous appendages regeneration. Taken together, these data provided new insights for the treatment of acute full-thickness wounds by cellular-material therapy and should contribute to new strategies for the treatment of chronic wounds and ischemic diseases.

## Conclusion

In this work, collagen scaffolds that were functionalized with a peptide with a specific affinity for MSCs were used in a porcine wound-healing model. After implantation at the injured skin, the functional scaffold effectively enhanced the recruitment of MSCs, accelerated the healing rate and promoted neovascularization. This study provided new insights for enriching autologous stem cells to the wound site and contributed to new methods for the treatment of wound healing.

## “Quick Response Code” Link for Full Text Artiles

The journal provides an easy and cool way to reaching to the journal’s website. Each article on its first page has a “Quick response code”. Using any mobile device with camera and internet connections, one can easily get to the full text of a particular article on the journal’s website. Start a QR-code reading software (or a tool included in an internet explorer) and point the camera to the QR-code in a journal, it will automatically take you to the HTML full text of that article.
